# Disturbance Frequency Determines Morphology and Community Development in Multi-Species Biofilm at the Landscape Scale

**DOI:** 10.1371/journal.pone.0080692

**Published:** 2013-11-26

**Authors:** Kim Milferstedt, Gaëlle Santa-Catalina, Jean-Jacques Godon, Renaud Escudié, Nicolas Bernet

**Affiliations:** INRA UR0050, Laboratoire de Biotechnologie de l'Environnement, Narbonne, France; Ghent University, Belgium

## Abstract

Many natural and engineered biofilm systems periodically face disturbances. Here we present how the recovery time of a biofilm between disturbances (expressed as disturbance frequency) shapes the development of morphology and community structure in a multi-species biofilm at the landscape scale. It was hypothesized that a high disturbance frequency favors the development of a stable adapted biofilm system while a low disturbance frequency promotes a dynamic biofilm response. Biofilms were grown in laboratory-scale reactors over a period of 55-70 days and exposed to the biocide monochloramine at two frequencies: daily or weekly pulse injections. One untreated reactor served as control. Biofilm morphology and community structure were followed on comparably large biofilm areas at the landscape scale using automated image analysis (spatial gray level dependence matrices) and community fingerprinting (single-strand conformation polymorphisms). We demonstrated that a weekly disturbed biofilm developed a resilient morphology and community structure. Immediately after the disturbance, the biofilm simplified but recovered its initial complex morphology and community structure between two biocide pulses. In the daily treated reactor, one organism largely dominated a morphologically simple and stable biofilm. Disturbances primarily affected the abundance distribution of already present bacterial taxa but did not promote growth of previously undetected organisms. Our work indicates that disturbances can be used as lever to engineer biofilms by maintaining a biofilm between two developmental states.

## Introduction

In macroscopic landscapes like forest ecosystems, disturbances as for example windfall may cause the onset of a new cycle of development [1]. The redeveloping forest goes through a succession of plant communities and their associated spatial arrangement. Battin et al. picked up the windfall example and used it in the development of biofilms in analogy to detachment after a flow-induced disturbance [2]. They argued that biofilms can be considered microbial landscapes formed by a response to environmental conditions and the interactions of their microbial inhabitants. The interplay between environmental conditions and microflora leads to the formation of an explicit spatial pattern of landscape elements (e.g. streamers or open patches) and corresponding microbial communities [3]. The emerging spatial pattern of landscape elements can only be observed at the landscape scale. Considering the length of streamers (e.g. up to three millimeters in a *Pseudomonas* biofilm [4]) and the size of open patches, the landscape scale may be on the order of millimeters to centimeters in many experimental or natural biofilm systems. When attempting to follow the development of the microbial landscape of a biofilm it is thus necessary to observe continuous areas that are larger than their landscape elements [5]. This is frequently impossible with traditional microscopic techniques and thus requires alternative analytical approaches. 

For biofilm ecology and equally for biofilm engineering, studies on the landscape scale are interesting for several reasons: a bird’s-eye view, for example on low-magnification images, may allow us to quickly classify the developmental state of a biofilm with tools developed for remote sensing. The morphological characteristics of a developmental state of a biofilm may be related to diffusive mass-transfer limitations and in consequence to rates of substrate turnover [6]. The availability of substrate will largely influence competitiveness of microbial inhabitants. Besemer et al. spearheaded work at the landscape scale on stream biofilms [7]. They demonstrated a correlation between landscape elements (base biofilm and streamers) and microbial communities at select time points. The temporal dynamics of the link between spatial organization of a biofilm and community composition remain a challenge for successful biofilm engineering and microbial landscape ecology.

Disturbances change microbial landscapes. This property of disturbances is often used deliberately as a lever for biofilm engineering when for example physical changes of the hydrodynamic conditions are used to detach excess biomass from bioreactors in wastewater treatment (e.g. 8). In the food industry or paper mills, chemical disturbances like the addition of a biocide are more commonly used to limit or stop biofilm growth [9]. Monochloramine is such a biocide. It is commercially important in drinking water treatment and treatment against biofouling of industrial sites (e.g. cooling towers or paper mills). In drinking water treatment, monochloramine or hypochlorous acid are often continuously dosed at a low level. The effects of this particular compound on biofilm growth have been studied extensively over the last years (see for example 10–12). Long-term consequences of continuous monochloramine exposure have been analyzed for example in reference [13]. There is experimental evidence that biofilm communities change as a response to continuous exposure to monochloramine [14] or hypochlorous acid [15]. In contrast, biofilm responses to recurring biocide exposure are less studied.

Sanderson and Stewart argue that discontinuous periodic shock loads may be more effective in controlling biofilm growth [16]. During discontinuous treatment, biofilms temporarily face high biocide concentrations, followed by periods without the presence of biocide. Unless the chemical treatment is followed by thorough physical cleaning, a remaining seed re-develops into a mature biofilm [17], much like a secondary succession that was observed in river biofilms [18]. Redevelopment necessitates recurring treatments. Consequently, in environments where long-term treatment of biocides is practiced, re-growing biofilms may develop resistance strategies [19] and change their response to recurring biocide pulse injection.

The long-term exposure of biofilms to recurring disturbances immediately prompts interesting questions but their analysis in natural ecosystem is often confounded by seasonal factors that influence biofilm growth as for example in natural river systems [20]. It may be advantageous to expose biofilm in the artificial but controlled environment of the laboratory to recurring disturbances. From a practical point, these investigations of biofilm response strategies to disturbances may offer alternative treatment procedures against biofilms in industrial settings. From an ecological point of view, a biofilm in the laboratory may serve as model to test how disturbance frequency shapes the observed biofilm landscape and its microbial inhabitants, as implied by Jessup et al. [21].

At the landscape scale of biofilms, limited information is available in the literature on disturbance responses and the link between morphology and community in microbial landscapes [22]. In this manuscript, we study the effect of disturbances on the biofilm landscape from a bird’s-eye view using low-magnification microscopy coupled with an image analysis approach first developed for aerial photography [23]. We relate for the first time the morphology development to the development of the bacterial community, quantified by molecular fingerprints at the landscape scale. It is obvious that our approach cannot resolve microbial interactions and the spatial organization of microbial communities at the scale of individual cells. We can, however, classify with our approach a biofilm based on the heterogeneous pattern of landscape elements – at the scale of the microbial landscape. The novel view that we offer in our work is related to approaches in remote sensing on global scales and complements the detailed knowledge on the microscale of biofilm development.

The biofilms in this study were exposed to recurring disturbances at two frequencies (i.e. daily and weekly pulse injections of the biocide monochloramine). We hypothesized that (a) frequent exposure to monochloramine pulses dampens the dynamic biofilm response by selecting a resistant community with an adapted biofilm morphology. (b) Less frequent disturbances provoke a dynamic response of morphology and community structure.

## Materials and Methods

### Reactor set-up and sampling

**Figure 1:**
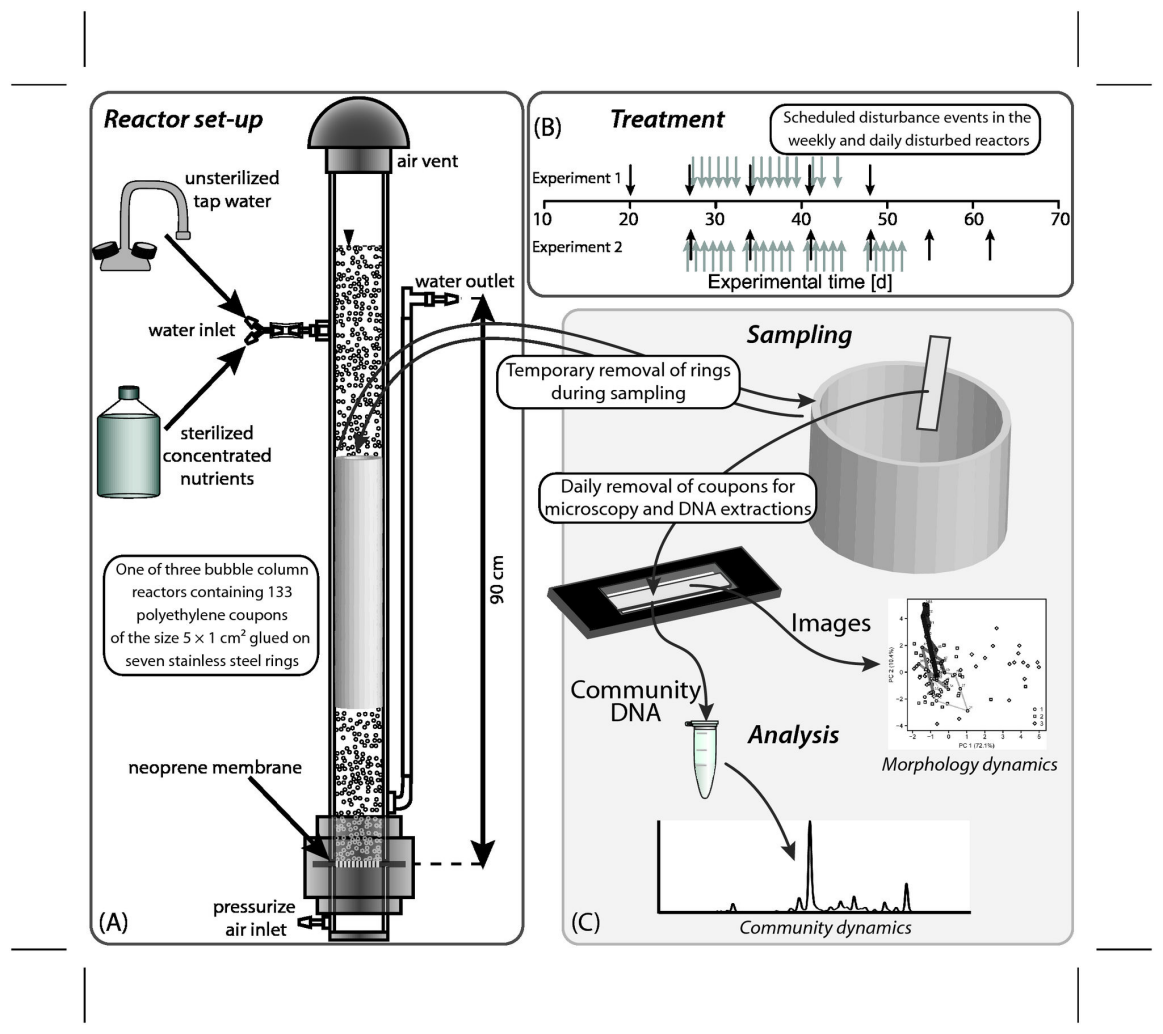
Experimental and analytical set-up of the reactor system. (A) The bubble column reactors: Aeration of the reactors served the purpose of providing oxygen, homogenizing the water column and applying a constant shear stress on the biofilm surface. (B) Monochloramine was added daily or weekly to two of the three reactors. (C) Biofilms were sampled by removing individual polyethylene coupons that were glued onto stainless steel rings. The rings were reinserted into the bubble column. On each coupon, biofilm morphology was analyzed by automated quantitative image analysis. From the same coupon, DNA was extracted and bacterial community dynamics were assessed using molecular community fingerprinting.

Open culture mixed biofilms were grown in three custom-made bubble column reactors in parallel in two replicated experiments over 56 and 70 days, respectively. The experimental set-up for one of the reactors is presented in Figure 1A. The polycarbonate columns of the reactors were filled with approximately 5 liters of water to a level of 90 cm. From the bottom of each reactor, compressed air at a rate of approximately 0.3 m^3^ h^-1^ was blown through a custom-made neoprene membrane of 2 mm thickness with 63 pores. Each pore was separated from its closest neighbor by approximately 1 cm. This set-up guaranteed non-limiting supply of oxygen and homogeneous substrate and shear conditions inside the reactors. The entire reactors were submerged in a water bath in order to maintain a temperature of 37±1°C inside the reactors. The water bath also prevented light exposure of the reactors. The experiment was replicated twice. Here, data from the first experiment are shown. The data for the second run are comparable and are presented in more detail in the supplemental materials.

In each reactor, seven stainless steel rings were submerged in the water column, closely lining the interior reactor wall. On the inner surface of each ring, 19 coupons with the dimensions 188 µm × 5 cm × 1 cm were glued with Master MS PRO (Master-In, Cagnes sur Mer, France) so that 133 coupons per reactor were available for sampling (Figure 1C). The coupons were cut from sheets of polyethylene foil used for cover slips (ibidi GmbH Integrated BioDiagnostics, Martinsried, Germany). 

During sampling, the stainless steel rings were withdrawn from the reactor and the desired number of coupons was detached, typically one coupon per reactor per weekday. The time interval between two samples was approximately 24 hours during the week and 72 hours over the weekend. After sampling, the rings were reinserted into the reactor. Sampling of the reactors took less than five minutes during which the biofilm was kept water-saturated. A protective foil at the bottom side of the coupon was removed with all remaining traces of glue, leaving a pristine surface on the backside of the coupon and the biofilm on the front side. The coupon was then placed in a custom-made holder that allowed us to keep it submerged in 600 µl of particle-free reactor effluent during microscopy and image acquisition. After imaging, the coupons were cut into three approximately equally sized pieces, each transferred into a 2 ml screw-cap vial and immediately stored at -20°C until DNA extraction. Typically, one of the three pieces was used for DNA extractions.

### Start-Up and Continuous Operation of the Reactors

Each of the three reactors was inoculated with a 100 ml aliquot of process water from a paper mill in a nutrient solution with the same composition as during continuous operation (see below) but at four times higher initial concentrations. Over the first 24 hours, the reactors were left in batch mode. During continuous operation, the reactor was fed sterilized concentrated nutrient solution through a syringe filter (0.2 µm, cellulose acetate) to prevent contamination of the nutrient reservoir via the reactor. The nutrient solution was prepared in autoclaved, deionized water and diluted by unsterilized tap water at a ratio of 1:75 at the inlet to the reactors. We used digital Masterflex L/S pump drives (Sodipro, Echirolles, France). The nutrient solution consisted of the following compounds at their respective inlet concentrations: Glucose (0.1 mM), yeast extract (1.9 g m^-3^), K_2_HPO_4_· 3 H_2_O (7 µM), (NH_4_)_2_SO_4_ (18 µM), NH_4_Cl (35 µM), MnSO_4_· H_2_O (0.07 µM), CuSO_4_· 5 H_2_O (0.05 µM), CoSO_4_· 7 H_2_O (0.02 µM), Na_2_MoO_4_· 2 H_2_O (0.02 µM), ZnSO_4_· 7 H_2_O (0.05 µM), and H_3_BO_3_ (0.05 µM) and FeCl_2_· 4 H_2_O (0.05 µM). A comparably short hydraulic retention time of 65 minutes was selected in the reactor to limit the presence and growth of suspended biomass. It was microscopically confirmed that the concentration of suspended biomass was negligible. With the ion-exchanger FMB 20V (CR2J aqualine, Croissy Beaubourg, France), part of the Ca^2+^ content of the tap water was replaced by Na^+^. Ion exchange was necessary to prevent the formation of calcium carbonate precipitates on the reactor surfaces. The dilution water was preheated using a TetraTec HT 300 heading rod for aquariums (Tetra GmbH, Melle, Germany). 

### Biocide preparation, dosage and quantification

The experiments were conducted in three phases: (1) initial biofilm development (three weeks); (2) repeated additions of the oxidative biocide monochloramine as pulse injections to two of the three reactors; (3) recovery without biocide addition in all reactors. In phase 2, we used injection frequencies of once a day for a period of 2.5 and 3.5 weeks in one reactor (15 and 22 additions) and once a week for a period of five and six weeks in the second reactor (five and six additions) in two replicated experiments (Figure 1B). The initial biocide concentration inside the reactors was adjusted to 10±2 g monochloramine m^-3^ expressed as Cl_2_. During the injection, dilution pumps were turned off for 30 minutes in all reactors to prevent immediate washout of the biocide in the dosed reactors and to expose the untreated reactor to comparable hydrodynamic conditions. Non-reacted biocide was washed out when dilution pumps were turned back on. Biofilms were sampled at least two hours before the biocide was added. This interval insured that potentially detached biomass from the reactor walls during sampling was washed out of the reactor and would not contribute a biocide demand. The first samples following a pulse injection were typically taken 15-18 hours after monochloramine exposure.

Monochloramine is not stable over time and was therefore prepared no longer than 30 minutes before the pulse injection from stock solutions of NH3· HBr (p.a.) (Merck, Darmstadt, Germany) and 12% technical NaOCl (BDH prolabo, Fontenay-sous-Bois, France) at a molar ratio of 1.2:1 at a pH of 9.3. The experimental conditions described in two patents were used for approximating optimal conditions during monochloramine synthesis in the laboratory. The patents form the basis for the application of monochloramine at the industrial scale [24–26].

Free chlorine and monochloramine were quantified using Hach powder pillows (Hach Lange, Marne La Vallée, France) and Hach methods 10069 and 10171, respectively, adapted for the use in 10 ml COD cuvettes with a ThermoFisher Helios Epsilon spectrophotometer (Fisher Scientific SAS, Illkirch, France). Also monobromamine that may have formed was quantified with this method.

### Microscopy and image analysis

A Leica M205FA stereomicroscope (Leica Microsystems GmbH, Wetzlar, Germany) equipped with a 0.63 × planapochromatic objective was used to acquire images on 14 locations per coupon covering its entire available biofilm area. At each location, two images were taken, one under transmitted and the second under oblique illumination. The two images per location describe different morphological features because of the difference in the illumination technique. Biofilm samples were submerged in particle-free reactor effluent for imaging without the need of fixation or staining. Images were acquired with a Peltier-cooled Leica DFC495 camera with a 2/3'' CCD chip and a maximum resolution of 8 MegaPixel. We used the software Leica ApplicationSuite (LAS) core V3.5.0 (October 2009) for taking images at 22 × magnification of the dimensions 2176 × 1632 pixels in 8-bit TIFF format. Each pixel has the dimensions of 2.92 µm, yielding an observation area of 6.4 × 4.8 mm^2^. The image resolution is sufficient to resolve the available optical information on the images. Quantitative image analysis based on gray level variations requires reproducible illumination conditions. We can rule out variability of illumination as an important error source in our experiments because we obtained reproducible illumination by calibrating the microscope each time for transmitted and oblique illumination with a custom-made reference.

High and low local biomass concentrations translated to dark and bright areas on transmitted light images because of light absorption. Areas with higher local biomass concentrations on oblique illumination images appeared brighter because of higher refraction of the inclined light beam. We validated our image analysis approach experimentally for transmitted light images in order to justify the interpretation of gray levels on biofilm images as the spatial arrangement of biomass. This validation is documented in Figure S5 in the supplemental materials to this publication. In Figure S2A and B, time series for average gray level development in the three reactors are shown. 

An in-house software coded in FORTRAN was used to calculate textural descriptors directly from the untreated TIFF images [27]. The approach uses spatial gray level dependence matrices (SGLDM, [23]). SGLDMs display histograms of gray level variation from multiple-pairwise comparisons of pixels. A defined separating distance between pixels in a given direction sets the rule for the comparisons. In this work we compared the gray level variation on all images between pixels in the directions of 0° and 45° off the image horizontal at distances of approximately 30, 60, 90, 120, 150, 180, 200, 230, 260, 290, 580, 730, 880, 1020, 1170 µm. Textural descriptors were then used to summarize the complex content of the SGLDMs. Textural descriptors derived from SGLDMs can be imagined as the equivalents of diversity indices calculated from molecular fingerprints. We used in this study the 15 textural descriptors from [27]. Altogether, a multidimensional data set with 900 variables was calculated for each of the 14 biofilm locations on each coupon: 2 angles × 15 distances × 15 descriptors × 2 illumination techniques. Each respective variable per location was averaged over the 14 locations to yield one set of 900 mean values describing the morphology of one biofilm sample.

We selected the SGLDM approach for the image analysis because the full gray level information contained in the images is used in the calculation. In other commonly used image analysis approaches, a binarization is necessary to differentiate biomass and void space. Consequently, the information that is contained in gray level variation is lost. At the larger spatial scale of our images (compared to traditional CLSM), this gray level variation is related to differences in local biomass concentration and should be considered. On the images at the landscape scale, void spaces hardly exist as essentially all available surfaces were covered by at least a thin biofilm.

### DNA extraction and SSCP

Biomass was detached by bead beating from one piece of the coupon with an area of 1.5 cm^2^. Community analyses were performed on only one out of three available pieces because unpublished preliminary experiments indicated that community structure on the three coupon pieces did not significantly differ from each other. For DNA extraction, the protocol in Rochex et al. [28] was followed, including a heat-treatment step. PCR-primers specific to the V3 region of the bacterial 16S rRNA molecule were used in a 50 µl PCR reaction with 36.9 µl molecular grade water, 5 µl of 10 × pfu turbo buffer, 4 µl of 2.5 mM dNTP mixture, 1.3 µl of forward primer (426 nM) and reverse primer (412 nM), 0.5 µl of 2.5 U/µl Stratagene PfuTurbo DNA Polymerase (Stratagene, La Jolla, CA, USA). PCRs were done using a Mastercycler epgradient S (Eppendorf AG, Hamburg, Germany). After an initial denaturation for 2 minutes at 94°C, 25 cycles of melting (1 minute at 94°C), annealing (1 minute at 61°C) and extension (1 minute at 72°C) were followed by a final extension at 72°C for 10 minutes. The (5’-3’) sequences for the forward- and reverse-primers are ACGGTCCAGACTCCTACGGG (*E. coli* position F331) and TTACCGCGGCTGCTGGCAC (*E. coli* position R500), respectively, as in [28]. The bacterial communities of the PCR-amplified DNA samples were analyzed in a 3130 genetic analyzer (AB applied biosystems, Carlsbad, CA, USA) using Capillary Electrophoresis-Single Strand Conformation Polymorphisms (CE-SSCP) under conditions described in [28]. A mixture of ROX internal size standards were added to each sample.

Bacterial fingerprints from the CE-SSCP profiles were aligned based on the ROX internal size standards and normalized using the package StatFingerprints in the software environment R [29,30]. A subsequent semi-automated binning procedure allowed us to further improve the alignment so that multivariate (PCoA) and multiple pairwise (Raup-Crick index) data analysis became more robust.

### Sequence identification of majority peak

Three samples were selected in which CE-SSCP profiles were dominated by the majority peak. These samples from day 21 in the untreated, day 50 in the weekly treated and day 44 in the daily treated reactors were PCR amplified using the forward- and reverse-primers GAGTTTGATCMTGGCTCAG (*E. coli* position F9) and TTACCGCGGCTGCTGGCAC (*E. coli* position R500), cloned using the pGEM-t vector system by Promega, (Madison, WI, USA) in DH10B competent cells (Invitrogen, Carlsbad, CA, USA), and subsequently Sanger-sequenced. CE-SSCP profiles were produced from the clones as specified above and compared to the environmental CE-SSCP profiles from which the clones were derived.

The taxonomy of the clone sequences was determined using the online RDP classifier [31]. The Basic Local Alignment Search Tool was used to recover similar sequences from the nt nucleotide collection of NCBI [32].

### Data analysis

All data analysis was made in the software environment R [30]. Image data from both experimental runs were combined in one data set, scaled and subjected to Principal Component Analysis (PCA) using the function rda in the R package vegan [33]. For the community analysis, two-dimensional Principal Co-ordinate analysis (PCoA) was done on binned CE-SSCP data from both experiments with a quantitative version of the Jaccard distance matrix as suggested by Oksanen et al. using the functions cmdscale and vegdist from the vegan package [34]. We analyzed image data and community data from both experimental runs in one respective PCA or PCoA so that data points in the figures for the two replicate experiments are comparable to each other. 

The –log Simpson diversity index was calculated with the aligned but unbinned CE-SSCP profiles using the R package StatFingerprints [29]. For this calculation, the use of unbinned CE-SSCP data is preferred because the microbial fingerprints are considered individually and therefore an optimized alignment by binning with its accompanying loss of data is unnecessary. The –log Simpson index takes into account community composition and abundance. In CE-SSCP profiles, composition is expressed as presence-absence of peaks and abundance as relative peak height. 

The Raup-Crick index was used to compare the similarity of community composition between subsequent samples (i.e. presence-absence of shared peaks between binned CE-SSCP profiles). The index can be interpreted as the probability that observed similarities (or dissimilarities) between communities are not different from similarities (or dissimilarities) between randomly assembled communities. For the calculation, the observed differences in composition are compared to differences between 9999 randomly assembled community pairs. All peaks that were ever detected in CE-SSCP profiles of the two experiments were made available for assembling the random communities. Raup-Crick indices smaller than 0 indicate higher observed similarities than expected between randomly assembled communities; indices greater than 0 point towards more different community compositions than expected by chance. Similarities or dissimilarities were considered significant when Raup-Crick indices were smaller than -0.95 or greater than 0.95, respectively. The index was calculated using the R code that Chase et al. provided on their website [35]. 

## Results

### Morphology and community dynamics before, during and after disturbances

**Figure 2:**
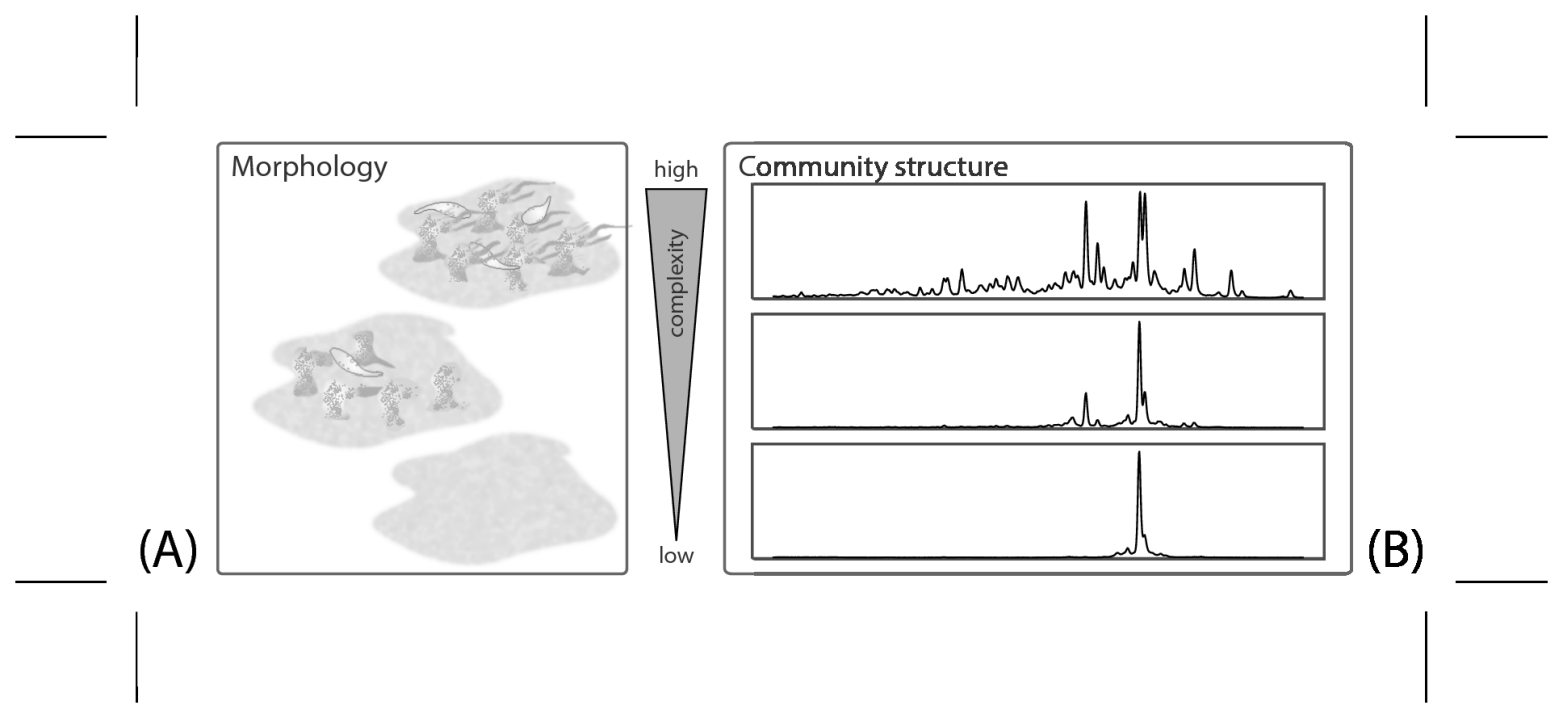
A description of the varying degrees of complexity for biofilm morphology and community structure. This description is used in the interpretation of Figure 3. (A) Slanted view on three stylized biofilms. A biofilms with a simple morphology is seen at the bottom; more complex morphologies are shown towards the top of the image. (B) Three exemplary community fingerprints as CE-SSCP profiles. Simple bacterial communities are dominated by one member, represented by one peak in the fingerprints. With increasing complexity, dominance of peaks in the profiles is less pronounced and peaks are more evenly distributed. The overall number of peaks increases.

During the periods indicated in Figure 1B, the oxidative biocide monochloramine was injected as a 30-minute pulse once a day or once a week. We quantified the effect of monochloramine on biofilm development and used Principal Components and Principal Coordinate axes with time to display the results. Principal Component and Principal Coordinate Analyses allow the graphical presentation of multivariate data in reduced dimensions. The first dimensions in our analysis captured a large portion of the variability contained in the multivariate signal from the images (PC 1 68% for the combined data of the two replicated experiments) and from the molecular fingerprints (PCoA1 69% for the combined data of the two replicated experiments). The meaning of the multivariate axes is not be directly accessible but requires a thorough interpretation as a large number of variables is condensed in few dimensions. We developed our interpretation during the careful visual examination of images and community fingerprints. Based on the observations, we synthesized in Figure 2 a visual definition of degrees of complexity for biofilm morphology and community composition. Figure 2 is meant to facilitate the interpretation of PC1 and PCoA1. We are aware of the simplification and subjectivity that accompany this interpretation but consider the increased accessibility of the multivariate axes an important benefit.

In Figure 2A, three biofilm morphologies of low (bottom), medium and high (top) complexity are stylized. In the experiments, the least complex biofilm morphologies were observed towards the end of the daily treatment periods. Biofilms at that time were dense layers of biomass of less than 100 µm thickness and homogeneous at the µm scale. Biofilms with a low complexity did not harbor protists visible with the stereomicroscope. The more complex the morphology became, the more heterogeneous the microbial landscape appeared: three-dimensional structures with maximum heights of more than 200 µm became common. These structures protruded into the bulk phase with a local thickness of twice the estimated average biofilm thickness.

 Biofilms at this level of morphological complexity were highly heterogeneous on the micro-scale because of less elevated features between the protruding structures. The areal density of ciliates increased to up to 20 individuals mm^-2^ and large numbers of unspecified flagellates appeared (Figure 2A top). The ciliates were identified to be of the order *Pleurostomatida* using the procedure in [36]. We interpreted images with low values of PC1 as biofilms with a more complex morphology. Biofilm images over two treatment cycles (Figure S1) and time series of some textural descriptors before PCA (Figure S2C-F) are included in the supplemental materials.

In analogy to the morphology analysis, we interpreted the PCoA1 scores for the molecular fingerprints as a measure for complexity of the community structure (Figure 2B). A correlation, even though weak, between the –log Simpson diversity index for the molecular fingerprints with their respective PCoA1 score (R^2^=0.48 for community samples from both experimental runs, Figure S4) supported our interpretation. Communities with low scores on the PCoA1 axis belonged to simple communities, frequently dominated by a single peak (Figure 2B bottom) that was present in CE-SSCP profiles from all reactors in both experimental runs. Communities with high scores on the PCoA1 scale correspond to molecular fingerprints with the largest number of peaks and the most even distributions of peak heights (Figure 2B top). In Figure S3, exemplary CE-SSCP profiles over three treatment cycles before PCoA are presented.

**Figure 3:**
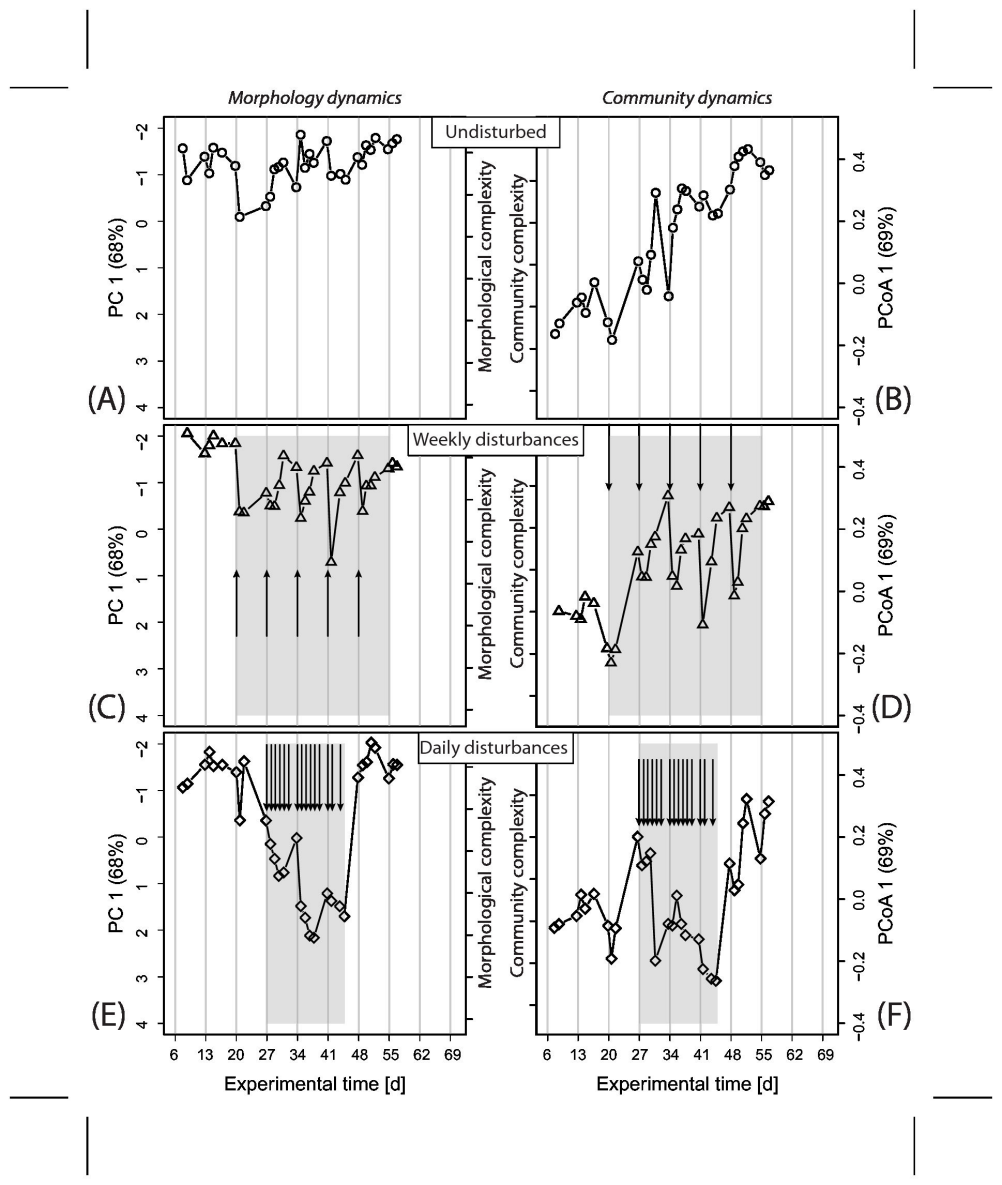
Morphology and community development with experimental time for the three reactors in the first experiment. The data for the second experiment can be found in Figure S6. Treatment periods were shaded where appropriate (panels C-F). Each monochloramine pulse injection is indicated by an arrow. Vertical gray lines mark Mondays. By performing Principal Component and Principal Coordinate Analyses, most of the variability in the data sets could be summarized in the first Principal Component (PC1) for morphology data and the first Principal Co-ordinate Axis (PCoA1) for the community data. More negative values on PC1 (note inverted axis labels) were related to more complex biofilm morphology. Similarly, the higher the value on PCoA1, the more complex was the community structure. Note the inverted axis in panels Figure **3** and S6A, C and E.

In Figure 3 and Figure S6, the temporal developments of PC 1 and PCoA1 for the three reactors in the experimental replicates are shown. In the first experimental run, the biofilms reached a comparably complex biofilm morphology with a simple community structure within six days of initial development. No samples were taken during this time. The morphology of biofilms in the second run was initially simpler (Figure S6). For the remainder of the experiment, the complexity of biofilm morphology as captured by PC1 remained comparably stable in the undisturbed reactors, especially in the first experimental run (Figure 3A). Biofilm communities in both runs were comparable to each other at all experimental states. We observed a trend of increasing community complexity in the undisturbed reactors until the end of the experiment. 

During the treatment period, the effects of weekly monochloramine pulse injections were immediately visible in samples following the pulse injections (Figure 3C and D, Figure S6C and D). Community structure was periodically simplified and redeveloped complexity over the remainder of the week. A similar behavior was detectable also for morphology data, especially in the first experimental run. 

 When monochloramine pulse injections were applied daily (Figure 3E and F, Figure S6E and F), the trend towards a simple morphology and community structure was stronger. Possible redevelopment between biocide pulses could not be observed during the treatment period as treatment and sampling occurred at the same frequency. Once treatment was stopped, community structure and morphology in the treated reactors quickly reached levels of complexity that were comparable to the untreated biofilm at the corresponding biofilm age. 

### Correlation between Morphology and Community Dynamics

**Figure 4:**
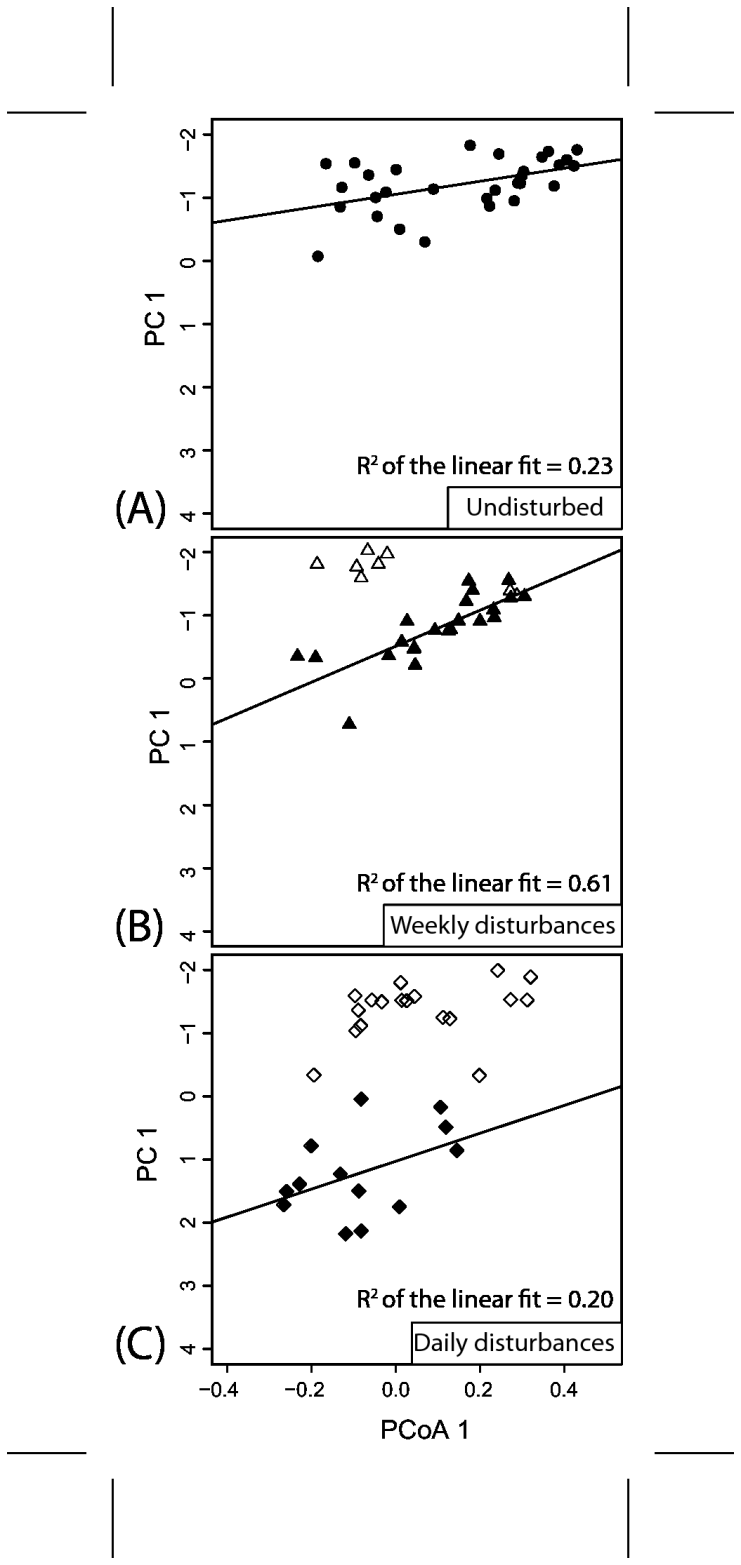
Linear regressions between morphology data (PC1 in Figure 3A, C and E) and community data (PCoA1 in Figure 3B, D and F). The regressions for the second experiment can be found in Figure S7. Only filled circles were considered for the regressions as these data were sampled during the treatment periods (or the entire experiment for the control reactor). Unfilled circles represent data taken before and after the treatment phases.

We correlated morphology and community dynamics for the two experimental runs in Figure 4 and Figure S7. The results for the two experimental runs were not systematic. We observed a relatively strong correlation during the weekly treatment phase in the first experiment (Figure 3C and D). It appeared that morphology and community dynamics were in phase during the recovery of the biofilm after the pulse injections. However, during the weekly treatment in the replicated experiment, the correlation between morphology and community data was weak and not significant. In this experiment, morphology and community data in the untreated reactor were most strongly correlated, while in the first one, only a weak (but significant) correlation was observed. Correlations during the daily treatment phase were weak and not significant in both experimental runs.

### What aspect of community structure was affected by monochloramine?

**Figure 5:**
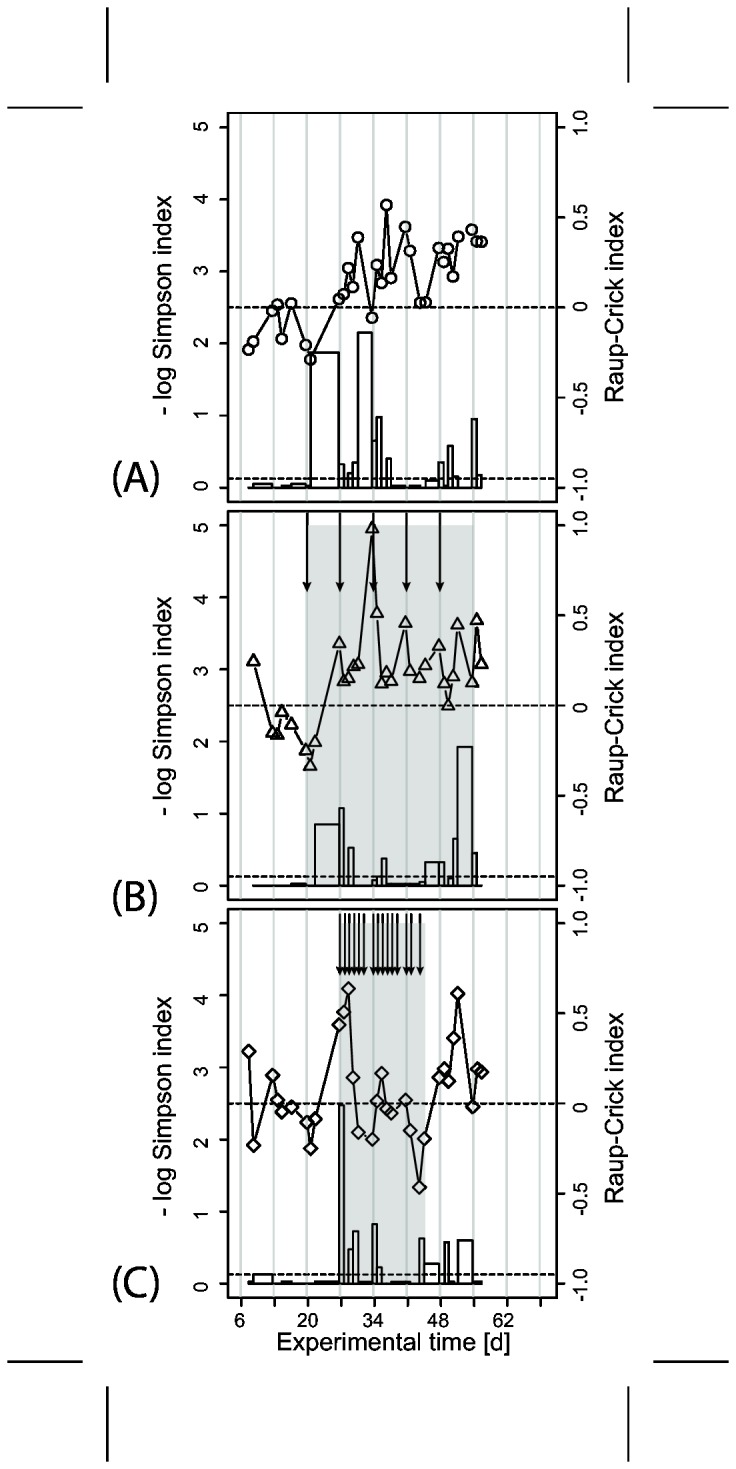
Development of bacterial diversity over time. We used the – log Simpson diversity index for individual molecular fingerprints and the Raup-Crick index to compare the composition (presence-absence of peaks) in a moving window of two subsequent samples from the same reactor. The solid lines with symbols represent – log Simpson diversity. The height of bars indicates the value of the Raup-Crick index for the comparison of the two samples at the left and right edge of the bars. Bar width stands for time between two samples and may vary. Raup-Crick values smaller than -0.95 indicate significantly more similar community compositions than expected from a random draw. Values around 0 indicate that observed differences could be explained by random effects while values greater than 0.95 indicate significant dissimilarities in composition. Shaded areas and arrows show treatment periods and monochloramine pulse injections. Dashed lines are the -0.95 and 0 levels for the Raup-Crick index.

Monochloramine pulse injections induced broad changes in the community structure as demonstrated in Figure 3. We investigated in more detail the change of two aspects of community structure: abundance patterns and community composition. In Figure 5 and Figure S8, we show the abundance- and composition-based –log Simpson diversity index over time and a pairwise comparison of subsequent samples using the Raup-Crick index. The Raup-Crick index is a probabilistic similarity metric based on presence-absence data and compares the difference in compositions between two samples to differences between two randomly assembled communities. The appearance of a rare community member therefore contributes as much to the change in community composition as that of dominant organisms. All observed values of the Raup-Crick index (with one exception) were below zero and can be interpreted as higher similarity between community compositions than expected from random communities. A large number of these pairwise-comparisons fell below the lower dashed line at -0.95 and were thus significant. The exception was one comparison after the daily treatment period in the second run with a positive Raup-Crick index, indicating a non-significant difference in community composition between two sampling dates. It appears that most of the elevated Raup-Crick indices coincide with a longer interval between the sampling dates. This implies that changes in community composition were more likely observed between samples that had time to develop between sampling dates.

During daily treatment, Simpson diversity decreased and reached an absolute minimum towards the end of the treatment (Figure 5C, Figure S8C). The Simpson diversity index in the weekly disturbed reactor decreased after each biocide pulse injection and subsequently increased to roughly previous levels with a local diversity maximum in samples before the next biocide pulse injection (Figure 5B). The highest absolute Simpson indices were measured for redeveloping biofilm between weekly pulse injections. The level of –log Simpson diversity for the untreated and weekly treated biofilm were comparable. Upon inspection of the CE-SSCP profiles, increases in Simpson diversity were mainly caused by increases in relative size of a number of already present minor peaks in the CE-SSCP profiles while the composition remained similar between samples. Decreases in Simpson diversity were related to increasing dominance of one already present peak in the profiles. Changes in Simpson diversity did not appear to be systematically related to changes in the Raup-Crick index. More elevated Raup-Crick values could be caused by minor fluctuations in rare members of the community that did not necessarily change the Simpson diversity of the samples. This is for example the case in Figure 5C at day 27. The highest observed Raup-Crick index in Figure S8C was indeed related to a drastically diversifying and changing community composition after the treatment period was stopped, visible also in the –log Simpson diversity. 

### Sequence identification of majority peak

The least complex CE-SSCP profiles in our sample collection were dominated by one single peak. We identified the sequence of the majority peak by cloning 16S rDNA fragments of the V1-V3 regions and comparing the CE-SSCP profiles from the clones with the experimental CE-SSCP profiles that contributed the DNA for cloning. Altogether 84 clone sequences were analyzed, represented by 27 unique sequence types. The most abundant sequence type was found 47 times. It dominated the clone libraries from all three samples. The second-most abundant sequence type was found five times. The CE-SSCP profile of the dominant clones aligned well with the majority peak in the experimental CE-SSCP profiles (Figure S9). The partial 16S sequence belongs with very high probability to an organism of the genus *Aquabacterium* and is identical to positions 21 - 513 of the sequence of an uncultured freshwater bacterium deposited to NCBI (accession number JF697431).

## Discussion

Two properties describe a microbial landscape: the bacterial community and the spatial arrangement of landscape elements like streamers, aggregated cells in microcolonies or void spaces. Using an automated image analysis approach and molecular fingerprinting, we were able to follow and compare the trajectories of morphology and community development in replicates of three independently operated laboratory biofilm reactors. After an undisturbed growth phase, common to all three reactors, recurring pulses of the biocide monochloramine were injected daily and weekly into two of the three reactors while one reactor served as control.

### Reproducibility of Biofilm Experiments

Surfaces in natural and engineered ecosystems are frequently colonized by a mature biofilm community that has developed over weeks, months or even years. The long developmental histories of naturally occurring biofilms are not necessarily reproduced in biofilm experiments that often only last several hours to days. In this study, we present data from two long-term biofilm experiments of 56 and 70 days. Long-term experiments over time-scales of weeks and months, however, come at the price of reproducibility between experimental runs as discussed in Lewandowski et al. [37]. At the same time, these authors emphasized the relevance of long-term studies because these biofilms represent more realistically maturing ecosystems. We were not able to replicate the initial morphological complexity in our two experiments (e.g. Figure 3A, C, E and Figure S6A, C, E). Biofilms in the first experiment reached a state of maximum complexity early on while biofilm in the second experiment showed a continuous development towards more complex morphology. In contrast to the morphology developments, the communities in both experiments behaved more similarly, leading to non-systematic correlations between morphology and community dynamics in our experiments (Figure 4, Figure S7). Development of morphology and community were tightly correlated in the untreated reactors in the second experiment but only weakly in the first (Figure 4A, Figure S7A). Similarly, morphology and communities during the phase of weekly treatment were correlated in the first experiment but not in the second (Figure 4B, Figure S7B). The inconsistent correlations between morphology and community structure on the landscape-scale in the two experiments were surprising. We expected a reproducible link between community structure and the formation of certain morphological features during biofilm maturation as observed in Besemer et al.’s biofilm systems [7]. 

Despite unavoidable differences in replicates of long-term biofilm experiments [37], the key results of our experiments were reproducible: The disturbance frequency determines the community structure and the morphology of a dynamically developing biofilm at the landscape scale. Even after repeated exposure to up to six weekly pulse injections of monochloramine, the biofilm reacted each time as after the first exposure. The development of an obvious resistance to the biocide was not observed.

### Disturbance frequency determines biofilm morphology and communities

The control biofilm and the two biofilms treated at weekly and daily intervals reproducibly developed unique characteristics during the treatment phase. Biofilms in the weekly disturbed reactors showed resilient behavior in terms of morphology and community development, oscillating between less complex states right after each monochloramine pulse injection and more complex states shortly before a new pulse injection. This behavior is especially pronounced in the resilience of the community structure. The exposure to monochloramine pulse injections removed prominent parts of the biofilm matrix by either direct oxidation or induced detachment. A loss of biomass after the treatments could be confirmed microscopically. A remaining base biofilm still contained the majority of community members as the high similarity in community composition before and after the disturbances demonstrated (Figure 5). 

In samples taken 15-18 hours after monochloramine exposure, we observed one distinct peak in the CE-SSCP profiles that had become more prominent. The peak indicates the presence of an organism of the genus *Aquabacterium*. Its increase can be interpreted as an increase in relative abundance of this strain of *Aquabacterium*, possibly masking the presence of rare community members in the CE-SSCP profiles. The resulting community structure was less even with a decreasing Simpson diversity while the community composition was maintained. In our situation, the community after a pulse injection may not be a subset of the healthy community as found for stressed communities in [38] but instead the same community with a shifted abundance distribution. The time between monochloramine exposure and the next sampling event possibly allowed the preferential regrowth of a surviving organism in the remaining base biofilm. Faster growth kinetics than the remaining community may thus give this organism a competitive advantage after the biocide pulse and lead to the observed simplification of the community. The simplification of morphology and community was reversed when slower growing organisms re-grew. The biofilm developed once more a complex morphology with time. It took approximately one week to recover a biofilm structure resembling the initial biofilm before the biocide addition. In the first experiment, the recovered biofilms were slightly more complex than before the disturbance. These observations may indicate a shorter time required to recover the initial community structure compared to the disturbance frequency. Stewart et al. illustrated the effect of disturbance frequency and time to recover biofilm thickness in a mathematical model [17]. Our experimental results for weekly and daily disturbed biofilms resemble the results that Stewart et al. obtained for fully recovered biofilms and biofilms that were recurrently disturbed when they reached their minimum thickness after a preceding disturbance [17]. In our experiments, it seemed that a biofilm could be held between two developmental states when the disturbance frequency matches the recovery time of a biofilm system. A wisely chosen disturbance frequency may thus be a lever to engineer a biofilm system to the needs for example in industrial processes.

When biocide pulses were injected daily into the reactor, the community structure and morphology became increasingly less complex over the treatment period. The least complex community fingerprints were dominated even stronger by the peak belonging to a strain of *Aquabacterium* that was already found in the weekly treated samples. In contrast to the weekly treated samples, only barely detectable rare peaks joined the *Aquabacterium* peak. Possibly, the 24 hours between two disturbances were long enough to allow regrowth of the *Aquabacterium* strain. The short recovery intervals compared to weekly pulse injections, however, increasingly penalized slower growing organisms over the faster growing bacterium.

**Figure 6:**
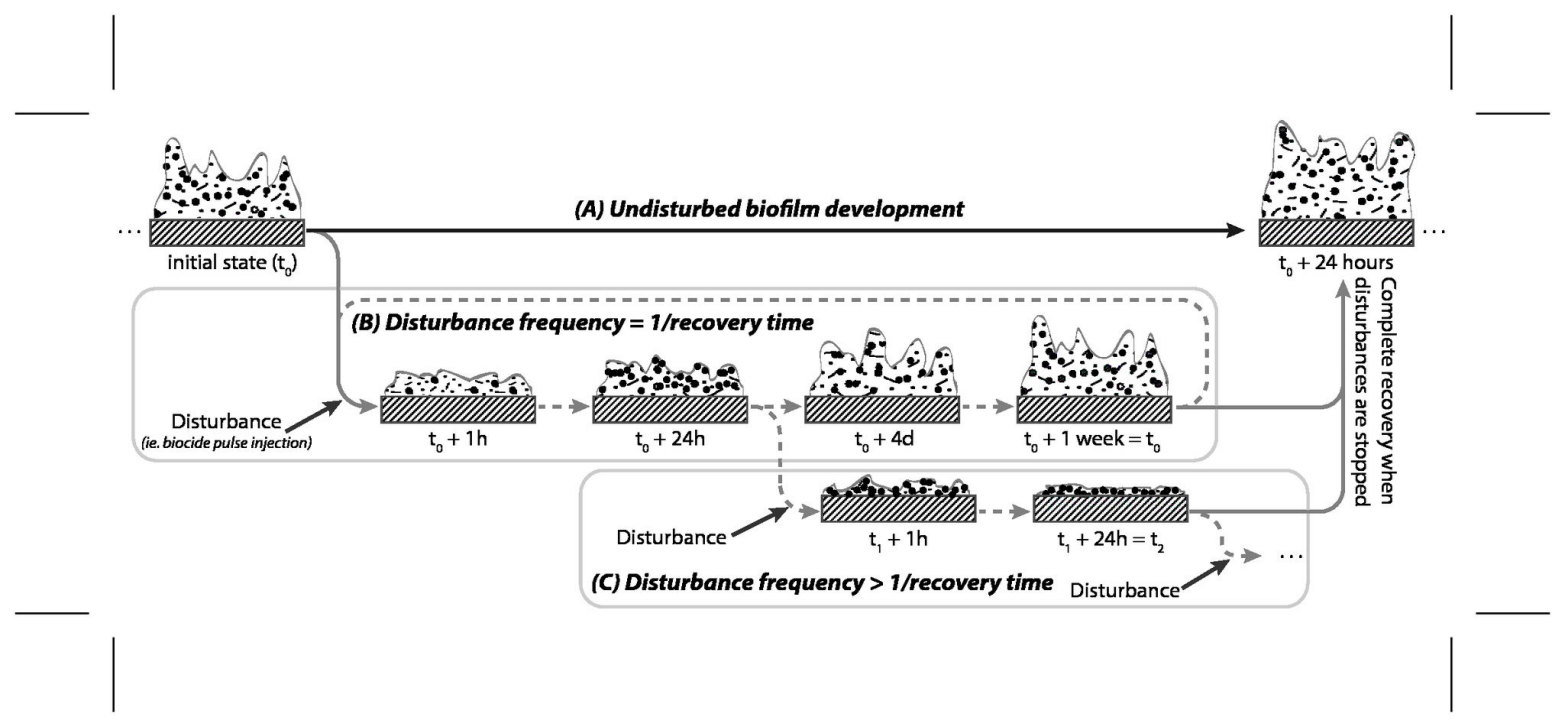
Effect of disturbance frequency (e.g. frequency of biocide pulse injections) on biofilm development. (A) Undisturbed development of a biofilm over 24 hours where t_0_ denotes the initial state. (B) A disturbance (here a biocide pulse injection) induces an indiscriminate loss of biomass, only affecting biofilm morphology. Subsequent regrowth initially favors fast-growing organisms (black points) while slower growing organisms regain importance after longer recovery. Recovery of its initial state is completed after approximately one week in our experiments. A recurring pulse injection induces another recovery cycle. If biocide pulse injections occur earlier than the recovery time (C), recovery remains incomplete and biofilm morphology and community structure tend to a state different from t_0_. Once disturbance pressure ceases, the previously disturbed biofilms regrow and approximate the undisturbed biofilm.

The highly disturbed reactors did not develop a stable biofilm structure even after four weeks of treatment but showed a trend of continuously simplification. It may be that the continuation of recurring pulse injections would have eventually led to complete washout of the biofilm. It is however equally imaginable that longer exposure to daily biocide pulse injections may yield a community that cycles between two states of morphology and community structure. We cannot investigate this hypothesis because more frequent sampling than done here is experimentally challenging in our current reactor setup. We have synthesized our findings into a conceptual model in Figure 6 that can be translated into a mathematical model to test the relation between disturbance frequency and cyclic development between two biofilm structures.

Once biocide pulse injections were stopped, the formerly weekly and daily treated biofilms quickly recovered a similar morphology and community structure comparable to the undisturbed biofilm. For the reactor treated at a high disturbance frequency, this resilient behavior was only seen once the treatment was over. The weekly treated reactor showed resilience already at the time-scale of the treatment frequency as the community recovered already between treatments. This resilience was not observed in the experiments by Roeder et al. [39]. Instead, these authors found a lasting long-term effect on the community structure of a biofilm after biocide exposure in an open biofilm system fed with unsterile tap water as in our case. It is imaginable that in our case, biofilms upstream of the reactor (e.g. the feed lines for tap water) or the tap water itself provided as a continuous seed community for the experimental biofilm. However, samples of biofilm communities in the distribution system of unsterile dilution water upstream of the reactor were different in composition from the recovering biofilm community in the reactors. It is therefore less likely that these biofilms were responsible for the immediate recovery of the observed community.

The quick recovery after the end of the treatment period confirms the notion that without proper physical cleaning, an unwanted biofilm will redevelop immediately once the disturbance pressure ceases. At the same time, disturbances like biocide pulse injections may be a useful tool to manage beneficial biofilms by controlling and maintaining their developmental state. Monochloramine could then be used not primarily for eradicating biofilms but rather for engineering biofilm development.

## Conclusions

• Observing biofilms at the landscape-scale [mm-cm] using low-magnification microscopy and community fingerprinting revealed the dynamic development of mixed-culture biofilm systems over several weeks.
• Weekly disturbed biofilms showed a resilient morphology and community at the time-scale of the disturbance frequency. A week-long recovery was sufficient to recover the initial biofilm morphology and community structure before recurring disturbances. Even after several weeks, biofilm responses to monochloramine were comparable and no adaptation became apparent.
• At high disturbance frequencies, biofilm morphology and community composition continued to simplify. A seemingly resistant bacterium of the genus *Aquabacterium* dominated the microbial community. Preferential growth of this bacterium after disturbance events may serve as explanation for the observed community dynamics.
• Within days, biofilm systems at both disturbance frequencies redeveloped a complex structure when disturbances were stopped.
• Monochloramine pulse injections could be used as lever to engineer morphology and community structure in biofilm ecosystems.

## Supporting Information

Figure S1 **A time-series of top view images of biofilms.** Images are acquired with opaque illumination before and after the second (days 27 - 31) and third (days 34 - 38) monochloramine pulse injection in the weekly treated reactor of the first experiment (Figure 3). Darker areas on the images contain less biomass than lighter areas. Monochloramine additions are indicated by an arrow. Each image covers a biofilm area of 6.4 × 4.8 mm^2^. The time interval between each of the images is approximately 24 hours. Most complex morphologies are seen before treatment (images to the left) and four days after the treatment (images to the right); least complex morphologies are observed on images immediately following the treatment. The contrast in the images was globally enhanced in order to improve the display on screen and in print.
(EPS)

Figure S2 **Six descriptors with experimental time for the three treatment scenarios in the first experiment (Figure 3).** The data were derived from images that were analyzed using the Spatial Gray Level Dependence Matrix (SGLDM) approach. Treatment scenarios are color-coded in each panel. Color-coded arrows indicate monochloramine pulse injections in the treated reactors. Panels A, D and E show results for images taken with transmitted illumination; panels B, D and F show results for images taken with oblique illumination. Mean gray levels from the images can be extracted by transforming the descriptor f6 (see 23): (f6/2)-1 = mean gray level. Mean gray level is independent of the angle-distance combination used in the analysis. Results for the descriptor Correlation (f3 in [23]) depend on these combinations. In Panels C, D and E, F, we contrast results that we obtained from the most different combinations that were used in our analysis (0° at a distance of 10 pixels (approximately 30 µm) for C and D; 45° at a distance of 400 pixels (approximately 1170 µm in E and F).
(EPS)

Figure S3 **A time-series of CE-SSCP profiles during the period of weekly treatment of the first experiment.** Arrows indicate the weekly pulse injections of monochloramine. Experimental time in days is given for each profile. A simplification of the profiles is noticeable after each pulse injection. Over the days following a disturbance, profiles diversify once more.
(EPS)

Figure S4 **Correlation of PCoA 1 scores with the respective –log Simpson diversity indices for both experiments.**
(EPS)

Figure S5 **Proof on concept for image analysis method.** Correlation between average gray levels from transmitted illumination images and gravimetrical measurements of biofilm biomass as total solids at the same sampling dates. Data from the first experiment described in this publication and an additional control experiment under identical conditions were presented in the figure. Error bars in x-direction indicated the standard deviations of 14 image repetitions. Error bars in y-direction indicated standard deviations of repetitions for total solids measurements that were done in three cases. The linear regression is flanked by 95% confidence intervals.
(EPS)

Figure S6 **Morphology and community development with experimental time for the three reactors in the second experiment.** Treatment periods were shaded where appropriate (panels C-F). Each monochloramine pulse injection was indicated by an arrow. Vertical gray lines mark Mondays. By performing Principal Component and Principal Coordinate Analyses, most of the variability in the data sets could be summarized in the first Principal Component (PC1) for morphology data and the first Principal Co-ordinate Axis (PCoA1) for the community data. More negative values on PC1 (note inverted axis labels) were related to more complex biofilm morphology. Similarly, the higher the value on PCoA1, the more complex was the community structure. Note the inverted axis in panels Figure 3 and S6A, C and E.
(EPS)

Figure S7 **Linear regressions between morphology data (PC1 in Figure S6A, C and E) and community data (PCoA1 in Figure S6B, D and F).** Only filled points in panels B and C were considered for the regressions as these data were sampled during the treatment periods.
(EPS)

Figure S8 **Development of bacterial diversity over time.** We used the – log Simpson diversity index for individual molecular fingerprints and the Raup-Crick index to compare the composition (presence-absence of peaks) in a moving window of two subsequent samples from the same reactors. The solid lines with symbols represent – log Simpson diversity. The height of bars indicates the value of the Raup-Crick index for the comparison of the two samples at the left and right edge of the bars. Bar width stands for time between two samples and may vary. Raup-Crick values smaller than -0.95 indicate significantly more similar community compositions than expected from a random draw. Values around 0 indicate that observed differences could be explained by random effects while values greater than 0.95 indicate significant dissimilarities in composition. Shaded areas and arrows show treatment periods and monochloramine pulse injections. Dashed lines are the -0.95 and 0 levels for the Raup-Crick index.
(EPS)

Figure S9 **CE-SSCP profiles of majority clones (profiles A-F) and low abundance clones (A'-C'** **)**.
At the top of the figure, reference profiles for biofilm samples from the first experiment are shown. DNA from these samples was taken for cloning.
(EPS)

File S1 **Integrates the supplemental figures in a text document.**
(PDF)
