# Supplementary material for: Disturbance Frequency Determines Morphology and Community Development in Multi-Species Biofilm at the Landscape Scale
Source: PLoS One. 2013 Nov 26;8(11):e80692. doi: 10.1371/journal.pone.0080692 (PMC3841191; doi:10.1371/journal.pone.0080692)
Supplement: File S1 — Integrates the supplemental figures in a text document. (PDF) [file pone.0080692.s010.pdf]

Supplementary Information

DISTURBANCE FREQUENCY DETERMINES MORPHOLOGY AND COMMUNITY DEVELOPMENT IN MULTI-SPECIES BIOFILM AT THE LANDSCAPE SCALE

K. Milferstedt, G. Santa-Catalina, J.-J. Godon, R. Escudié, N. Bernet

INRA, UR0050, Laboratoire de Biotechnologie de l'Environnement, Avenue des Etangs, Narbonne, F-11100, France

Corresponding author: Kim Milferstedt (kim.milferstedt@supagro.inra.fr)

Manual vs. automated analysis of microscopic images

A direct visual interpretation of time series of images from biofilm experiments requires experience and is frequently difficult to communicate on printed images or images displayed on a monitor. The images in Figure S 1 illustrate this dilemma, as most readers will find a meaningful differentiation of the images difficult. The automated image analysis approach, however, offers a less subjective alternative as the data in Figure 3 in the main manuscript and Figure S 6 demonstrate.

Raw data from the image and community analyses

Presenting image analysis data and community fingerprints in ordination plots allows us to summarize a multivariable dataset in few dimensions. In Figure S 2 and Figure S 3, we present some examples of raw data from the image and community analysis to allow a better appreciation of the data.

Relating community complexity to microbial diversity

The definition of community complexity that we developed in Figure 2 is supported by the relation of PCoA 1 to the –log Simpson diversity of the CE-SSCP profiles. This correlation is shown for data from both experiments in Figure S 4.

Confirming the validity of the image analysis approach

The image analysis approach that we used requires an experimental validation in order to justify the interpretation of gray levels on biofilm images as the spatial arrangement of biomass. This validation consists of the correlation between the fundamental parameter “biofilm biomass” (measured as total solids) and average gray level as the most basic property on transmitted illumination images. We validated the approach during the first experiment and present the correlation in Figure S 5. In the present case, the difficulty of this correlation is the limited amount of available biomass on each coupon. Biofilm biomass is on the order of 10 milligrams per coupon and therefore pushes the method close to the detection limit of conventional analytical balances. This introduces a non-negligible source of error in the gravimetrical measurement of total solids. We were thus forced to pool biomass from various coupons in order to reach a reliable measurement. Considering the biomass limitation, the relatively weak correlation in Figure S 5 is acceptable as proof of concept for the interpretation of gray levels in terms of accumulated microbial biomass in a biofilm.

The quantitative analysis of biofilm images based on gray level variations is rarely done in the literature despite its ease and its usefulness especially when dealing with large numbers of images with areas of several mm^2. More commonly, biofilms are fluorescently stained prior to imaging at the μm scale using confocal laser scanning microscopes. At the typical scale of confocal laser scanning microscopy, the landscape nature of the biofilm may not be captured. As a tradeoff, the results of our method for image acquisition and the automated image analysis approach cannot be directly pointed towards a specific feature of biofilm morphology. Rather, PC1 should be interpreted as a continuous scale that classifies landscape morphologies. It should, however, be noted that our image analysis approach has its limits. Naturally, it is impossible to completely describe and differentiate the complexity of a time series of biofilm images in one dimension, even though PC1 is already composed of a wide range of information. Despite the intangible information content of PC1, the ability to reasonably classify biofilm images based on their displayed texture merits the use of our approach. The correlation between gray levels and biofilm biomass indicates that the approach is applicable also in the context of our experiments.

Experimental replication

We have replicated the experiment twice. In the main body of the manuscript, we discuss data from both experiments but only show results for the first experiment. The equivalent data for the second experiment are displayed in comparable figures in this section of the supplemental materials.

Identifying sequence for majority peak

In Figure S 9 we show three referenc CE-SSCP profiles from samples taken during the first experiment. These samples were taken for a cloning experiment. CE-SSCP profiles A-F represent clones with identical sequence. This sequence type dominated the clone libraries from each biofilm sample. The peak positions of the majority clone coincide with the majority peaks in the biofilm samples. CE-SSCP profiles A'-C' belong to sequences types at low abundance in the clone libraries and demonstrate the variability of fingerprints as a function of DNA sequence.
